# Efficacy of a brief multifactorial adherence-based intervention on reducing the blood pressure of patients with poor adherence: protocol for a randomized clinical trial

**DOI:** 10.1186/1471-2261-10-44

**Published:** 2010-09-27

**Authors:** Alfonso Leiva, Marta Fajó, Luís Escriche, Francisco J Audera, Sara López, Ma Carmén Martín, Rosa González, Gaspar Tamborero, Elena M García, Rosa Duro, Ramón Orueta, Francisca Serra, Pilar D'agosto P, Jerónima Miralles, Patricia Lorente, Joan Llobera, Ana Aurelia Iglesias, Ruth Fernández, María M Colom, Aina M Buades, Lucía Moreno, Clara Vidal

**Affiliations:** 1 Primary Care Research Unit of Mallorca, Baleares Health services-IbSalut, CAIBER, Mallorca, Spain; 2 Faculty of Science, Microbiology and Public Health Department, Zaragoza University, Zaragoza, Spain; 3 Son Ferriol Health Centre, Baleares Health services-IbSalut, Mallorca, Spain; 4 Monzón Health Centre, Aragon Health services-SaludAragón, Huesca, Spain; 5 Actur Sur Health Centre, Aragon Health services-SaludAragón, Zaragoza, Spain; 6 Coll D'en Rabassa Health Centre, Baleares Health services-IbSalut, Mallorca, Spain; 7 San Agustín Health Centre, Baleares Health services-IbSalut, Mallorca, Spain; 8 Santa María Health Centre, Baleares Health services-IbSalut, Mallorca, Spain; 9 Silleria Health Centre, Castilla la Mancha Health services-SESCAM, Toledo, Spain; 10 Sineu Health Centre, Baleares Health services-IbSalut, Mallorca, Spain; 11 Son Serra Health Centre, Baleares Health services-IbSalut, Mallorca, Spain; 12 Department of pharmacy, Baleares Health services-IbSalut, Mallorca; 13 Biescas Health Centre, Aragon Health services-SaludAragón, Huesca, Spain; 14 Sub-direction of nursing, Baleares Health services-IbSalut, Gabinete atención primaria, Mallorca, Spain

## Abstract

**Background:**

Lowering of blood pressure by antihypertensive drugs reduces the risks of cardiovascular events, stroke, and total mortality. However, poor adherence to antihypertensive medications reduces their effectiveness and increases the risk of adverse events. In terms of relative risk reduction, an improvement in medication adherence could be as effective as the development of a new drug.

**Methods/Design:**

The proposed randomized controlled trial will include patients with a low adherence to medication and uncontrolled blood pressure. The intervention group will receive a multifactorial intervention during the first, third, and ninth months, to improve adherence. This intervention will include motivational interviews, pill reminders, family support, blood pressure self-recording, and simplification of the dosing regimen.

**Measurement:**

The primary outcome is systolic blood pressure. The secondary outcomes are diastolic blood pressure, proportion of patients with adequately controlled blood pressure, and total cost.

**Discussion:**

The trial will evaluate the impact of a multifactorial adherence intervention in routine clinical practice. Ethical approval was given by the Ethical Committee on Human Research of Balearic islands, Spain (approval number IB 969/08 PI).

**Trial registration:**

Current controlled trials ISRCTN21229328

## Background

The prevalence of hypertension is about 35% for adults in general, about 40% for middle-aged individuals, and up to 68% for elderly individuals [1-3]. Most population-based studies confirm that hypertension increases the risk for cardiovascular events. For example, a recent meta-analysis indicated a continuous, strong, and graded relationship between blood pressure (BP) and the occurrence of atherosclerotic events [4]. Numerous antihypertensive agents can effectively lower BP and significantly reduce stroke, coronary heart disease, cardiovascular death and total mortality by 30-40%, 20-21%, 26-28%, and 13-16% respectively [5]. However, in clinical practice, hypertension can be difficult to control. It is estimated that only 40% of primary care hypertensive patients have BP below 140/90 mmHg, or below 130/80 mmHg for patients with diabetes or renal failure [6]. Clinical inertia and low adherence to medication are considered the major modifiable causes of poor BP control [7,8]. Adherence can be defined as the extent to which patients follow the instructions they are given for prescribed treatments. The prevalence of low adherence to antihypertensive drug therapy (AHT) has been estimated as 30-50% [9,10], and patients with poor adherence typically experience increased BP [11]. A report by the World Health Organization (WHO) addressed the general importance of improving adherence to long-term medical treatments [12]. The WHO report concluded that adherence is the result of a complex interaction of the social environment, patient, and healthcare professionals. It has also been reported that low adherence can increase the cost of treating hypertension by 15-20%, and is associated with more frequent hospitalization, use of emergency services, and admission to intensive care [13].

It has been shown that low adherence to long-term treatment often occurs when the treatment is complex or when the disease is asymptomatic (such as hypertension). Low adherence to AHT leads to reduced drug effectiveness and increased risk for the development of cardiovascular morbidities [10]. Moreover, adherence to AHT has been reported to reduce the risk of hospitalization by about 50%, and is also associated with reduced mortality [14,15].

Previous studies have reported that the poor adherence to AHT is due to patient perception that the disease is not significant, adverse drug effects, lack of treatment effectiveness, and the patient's poor or incomplete knowledge of the disease [16,17]. Clinical trials have shown that several strategies aimed at improving adherence to AHT were effective. A systematic review [18] indicated that the most effective interventions were simplification of dose regimens, reminder packaging, social and family support, BP self-registration, follow-up phone calls by nurses, teaching self-determination to patients, and graphical reminders for taking medication. Additionally, the use of multiple interventions is more effective than use of a single intervention.

The motivational interview, defined as a clinical patient-centered interview that helps to investigate and resolve ambivalence in unhealthy behaviors and/or habits to promote changes toward healthier lifestyles, is a recent method that can be used to promote behavioral changes in patients [19]. This process is more likely to be successful if the patient has a positive attitude about the need for change. Motivational interviewing is intended to help patients recognize and address their problems and to enhance their perception of treatment efficacy [20]. Optimal adherence to AHT depends largely on the relationship established between the healthcare professional and patient [21]. If the healthcare professional merely issues unilateral declarations about a treatment plan, the patient is less likely to adopt changes that have a positive impact on his/her health [22]. The motivation for change is greater if a patient and his/her healthcare professional work together to make treatment decisions. Additionally, patient outcome is better if the patient assumes responsibility for his/her own circumstances (locus of control) [23]. Clinical interviewing has been shown to be effective in the treatment of alcohol problems, drug addiction, smoking, obesity, and insufficient physical exercise.

The purpose of the present study is to evaluate the effectiveness of a multifactorial intervention that is designed to improve the systolic and diastolic BP of patients who have low adherence to AHT and elevated BP.

## Methods and Design

### Overview

**Figure 1:**
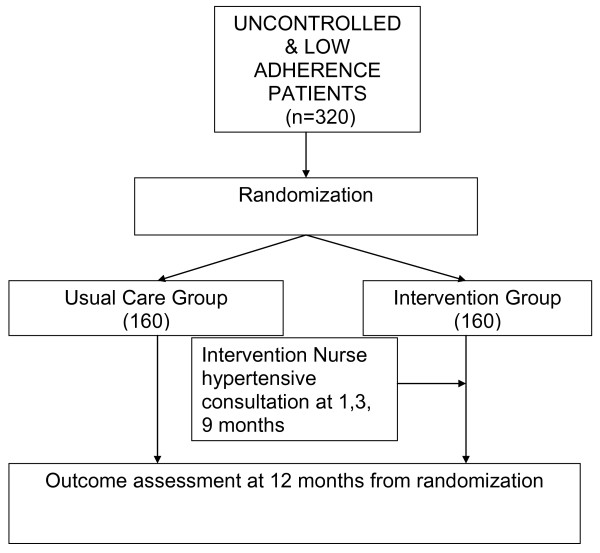
**Study design Flow-chart**.

Patients in a primary-care setting who have had low adherence to AHT, as assessed by the Morisky-Green or Haynes-Sackett tests, and elevated BP will be inform and ask to sign informed consent, those patients that agree to participate will be randomized equally to a "multifactorial intervention" group or a control group (which continued the usual care) (Figure 1). Participants in the intervention arm will receive three intervention sessions during a 9-month period. The intervention will be provided by study nurses who will have received special training for administration of motivational interviewing and in the implementation of the study protocol.

Table 1 summarizes the measures and variables according to the timeline. Participants will be studied for 1 year following randomization. Primary and secondary outcomes will be assessed by an external nurse who will be blinded to treatment assignment after 12 months. The primary outcome will be systolic BP. Secondary outcomes will include diastolic BP, direct costs, and the number of patients who required treatment to yield one patient with normalized BP.

**Table 1:** Measures, variables and timeline

VISIT	-1	0	1	2	3	4
Time (months)	-1	0	1	3	9	12
Blood pressure measurement inclusion criteria	X	X				
Morisky-Green and Haynes-Sackett tests	X					
Inclusion and exclusion criteria	X					
Comorbidities		X				
Blinded blood pressure measurement						X
Medication use						X
Adverse effects to medication		X				X
Medical consultation, cardiovascular events and hospital admission.						X
Checklist and time duration of intervention components (Intervention group)			X	X	X	
Motivational interview (Intervention group)			X	X	X	
Pill reminder (Intervention group)			X			
Family support (Intervention group)			X	X	X	
Blood pressure self-recording (Intervention group)			X	X	X	
Simplification of dosing regimens (Intervention group)			X			

Information bias will be minimized by use of a design that provides the maximum possible level of masking for studies of non-pharmacological intervention. In particular, the outcome assessor, data analyst, and physician will all be blinded to patient allocation. Misclassification, if any, is expected to be non-differential. Quality control will include recording of patient adherence to interventions, inclusion and follow-up monitoring, and quality supervision of data entry. We will perform an intention-to-treat analysis of the data; that is, the analysis will be by randomized assignment regardless of participation in intervention sessions.

### Study population

This study will enroll 320 patients, 18-75 years of age, who are being treated with AHT(s), remain hypertensive, and have low adherence to AHT as assessed by the Morisky-Green or Haynes-Sackett tests.

### Inclusion and exclusion criteria

The inclusion criteria are: age 18-75 years; poor BP control based on European Societies of Hypertension and Cardiology (ESH/ESC) guidelines (< 140/90 mmHg or < 130/80 mmHg in patients with diabetes or renal failure); and low adherence to medication as assessed by the Morisky-Green or Haynes-Sackett tests. The exclusion criteria are: presence of non-essential hypertension; treatment by hemodialysis; and institutionalization or terminal illness.

### Randomization to treatment groups

The randomization sequence will be computer-generated, stratified by nurse and blocked in groups of two. Study nurses will call the research units to give patient identification numbers and will be told the randomly assigned patient allocations. After this, participants in the intervention group will be scheduled for their first intervention visits.

The outcome assessors, data analyst, and physician will be blinded to patient allocation. To evaluate the effectiveness of blinding, these individuals will be asked to choose the arm to which they believed each patient was assigned (possible answers: intervention, usual care, or unknown). If they answered "intervention" or "usual care", they were asked to indicate what led to that belief.

### Multifactorial Intervention

The intervention consists of five components that will be delivered during three interventional visits of about 30 min each. Use of a detailed intervention protocol and comprehensive motivational interview training is intended to increase the effectiveness of interventions. The study nurses will carefully document each component of the multifactorial intervention that they perform by use of specially designed intervention visitation documents and will register the time and the duration of each component of the intervention.

The five components of the intervention are:

1. ***Motivational interview: ***This will be based on the Health Belief Model [24] and the Prochaska and DiClemente stage of change [25], and will try to resolve ambivalences that patients have regarding medication and explore perceptions that patients have about their ability to control events (locus of control).

2. ***Pillbox Reminder: ***A pillbox organizer with seven compartments will be given to the patients. They will self-fill it with one pill for each day of the week. Use of the pillbox will be explained to patients in the intervention group.

3. ***Family Support: ***Patients will designate one person in their household who will be responsible for reminding and monitoring medication intake. The designated person must accept responsibility of reminding the patient to take medication and of supervising pill intake. If a patient lives alone or does not wish to involve a family member or carer, they do not need to designate anybody. Thus, this part of the intervention is not mandatory.

4. ***BP measurements and AHT reminder forms: ***Twelve forms (one per month) and a table will be completed by the patient and nurse and used to record the date and hour of all BP readings (including gaps). AHT dosage and a telephone contact number will also be recorded on each form. This information will be frequently updated. The forms will be provided to patients and nurses at visit 0, and patients will be asked to bring them to all subsequent visits.

5. ***Simplification of dosing regimens: ***AHT dosage will be recorded at visit 0, and pharmacists will simplify the dosing regimen when possible at subsequent visits.

Visit 1 (first month, 40 min total): motivational interviewing (25 min), pill reminder (5 min), self-recording of BP (5 min), family support, when possible (5 min).

Visit 2 (third month, 35 min total): motivational interview (25 min), simplification of dosing regimen, if possible, check of self-recording BP (5 min), family support, when possible (5 min).

Visit 3 (ninth month, 35 min total): motivational interview (25 min), check self-recording BP (5 min), and family support, when possible (5 min).

### Control group

Patients randomized to the control group will not receive any changes in their care. However, they will be contacted at baseline and at the 12th month and asked to complete the same outcome measures as the intervention group.

### Training

The intervention nurses will attend a 16-h motivational interview training program to teach the theoretical underpinnings, principles, and "spirit" of motivational intervention and practical exercises (to be supervised by co-investigators Rosa Duro and Alfonso Leiva). This training program will include a thorough discussion of the study protocol and will instruct nurses to use terms consistently when recording the findings and documenting treatments on the intervention visitation documents. There will also be hands-on training sessions to ensure that the intervention nurses are comfortable with the protocol and the types of questions that participants may ask. The intervention nurses will also practice completing intervention forms and providing suggestions for self-care.

### Assessment of outcomes

#### Primary outcome measure

The primary outcome measure is systolic BP at 12 months. BP will be measured according to ESH/ESC guidelines. Patients (instructed to not eat or smoke for 30 min) will be seated quietly, with their backs supported and feet on the floor for 5 minutes. Then, using an appropriately sized cuff, the bared arm will be supported, and an oscillometric BP machine with a printer will be used for three measurements. The nurse (blinded to patient allocation) will write all three BP measurements on the final visit form.

#### Secondary outcome measures

Secondary outcomes are diastolic BP, proportion of participants with adequate BP control at 12 months, total direct cost, absolute and relative risk reduction, number of patients need to treat for benefit, and change in adherence at 12 months (measured by the Haynes-Sackett test).

### Statistical Issues

#### Sample size, clinical significance, statistical significance

We want to ensure that our clinical trial has adequate statistical power to detect a clinically significant difference of at least 6 mmHg at 1 year in systolic BP between groups with a two-tailed significance level of 0.05. Assuming a standard deviation of 15 mmHg and a 20% loss to follow-up, we would need to randomize 110 participants per group. We expect a 15% "contamination" of the control group (see below), yielding a 1.41 contamination effect [26]. Thus, the final target sample size is 320 patients.

The effectiveness of the intervention will be assessed by a clinically significant reduction in BP, cardiovascular mortality models shows an estimated 5-yr risk reduction of cardiovascular disease of 15-20% by a reduction of 5 mmHg in systolic blood pressure [27,28]. We have estimated that a difference of at least 10 mmHg at 1-yr is necessary to detect a clinically significant difference of at least 5 mm Hg at 5-yr.

Intervention will be provided to a group of participants by a single nurse, so we will consider the cluster effect during statistical analysis.

#### Data collection and management

We will collect information on outcome at every stage of the recruitment, randomization, treatment allocation, follow-up, and analysis so that we can report patient flow according to CONSORT guidelines [29]. We will record the number of patients who qualified for inclusion but who were not willing to participate, the number of patients assigned to the intervention arm, the number of patients assigned to the control arm, the number of intervention visits, the number of patients who provide follow-up data, the number of patients who complete the trial and are included in the analysis, and the number of withdrawals.

We will implement procedures to ensure that randomization proceeds as planned, that clinical data collection forms are accurately entered into databases, and that data are accurately transferred and retrieved as needed. We will use a relational database to track information during recruitment, randomization, treatment, and assessment so that we can report patient flow automatically and in an integrated fashion by use of standard automated reports. To ensure accurate transfer of information, all data system processes will be thoroughly tested prior to recruitment. Procedures to protect the confidentiality and integrity of the databases and documents will be reviewed by the biostatistician prior to patient recruitment and periodically during the study. To maintain the confidentiality of patient information, all patients will be identified by study numbers. The password security system will be used to assign appropriate levels of computer privileges to different database users to ensure that all personnel remain blinded. Computer files with patient names will be password-protected, with access restricted to staff who use this information to recruit patients, obtain follow-up data, and interact with patients who report adverse events.

During the intervention phase of the trial, we will continuously monitor the nurses' interventions and provide focused supplemental training as needed. After auditing to ensure adherence to the protocol, initials from the charts will be removed and stored in locked filing cabinets that are only accessible to study personnel. All data analysis files will be password protected.

### Protection of human subjects and assessment of safety

Our study protocol was approved by the Primary Care Research Committee and Mallorca Ethical Committee of Clinical Research (IB 969/08 PI).

### Adverse Events

Participants will be asked about adverse events at each visit. We define an adverse event as any unfavorable or unintended sign, symptom, or disease that could reasonably be associated with the use of AHT. If a patient develops a serious adverse event (death, a life-threatening event, inpatient hospitalization or prolongation of existing hospitalization, persistent or significant disability/incapacity, cancer, or fetal exposure) during the course of the study, it will be reported to the Spanish Agency of Medication Registry for Drug Adverse Events.

### Statistical Analysis

The primary statistical analysis will determine if our multifactorial intervention to improve AHT adherence is effective in lowering systolic BP and if the intervention is cost-effective. We will use intention-to-treat analysis for all data analyses; that is, all randomized patients will be included in the analysis, regardless of participation in any treatment sessions. This approach reduces the bias that may occur when participants who do not receive assigned treatments are excluded from analysis. All tests will be two-sided and α-values of 0.05 will be considered statistically significant.

We will test for significant differences between baseline characteristics of control and intervention groups. We will perform descriptive analysis, with continuous variables summarized by their means and standard deviations for normal distributions, and by median and 25^th ^and 75th percentiles for non-normal distributions.

In bivariate analysis, we will compare systolic and diastolic BP between the intervention and control groups at visit 4 (12th month) using the Student's *t*-test if both sample groups had normal distributions or the Mann-Whitney U test against the usual null hypothesis of no difference between means if the sample groups had non-normal distributions. We will also calculate 95% confidence intervals to assess the clinical significance of our treatment.

In multivariate analysis, we will adjust for clinical inertia (physicians blinding and number of medication changes) in a multiple linear regression model.

We will test for a significant difference in the percentage of patients with controlled BP (according to ESH/ESC guidelines) in the intervention and control groups using the χ^2 ^test. We will estimate relative and absolute risk reduction and the number needed to treat, defined as the estimated number of patients who need to be treated with the intervention (rather than the usual care) for one additional patient to be controlled. The 95% confidence intervals for these calculations will be provided. The number needed to treat is calculated as the reciprocal of the difference between the proportion of patients controlled in the intervention group and the control group.

An analysis of direct costs and outcomes for patients in both groups will be conducted by a nurse blinded to patient allocation. Clinical record review and detailed self-reported data on costs of healthcare utilization, clinical tests, and medication will be considered. All resources utilized will be multiplied by the appropriate cost using nationally applicable cost data. The cost of resource utilization (primary care visits, hospital admissions, clinical testing) will be estimated using the healthcare costs published in the *National Official Journal of Spain *or *Regional Officials Journal*. Costs of all AHTs will be calculated using prices published in the catalogue of pharmaceutical compounds by the Spanish Board of Pharmacists. Intervention costs will also consider the time needed to train nurses and the time of each intervention visit.

The primary health outcome for the cost analyses is BP reduction, which will be calculated using direct costs. The measure of "relative value for money" from the intervention is the incremental cost-effectiveness ratio calculated from the additional cost for a benefit of 1 mmHg reduction.

Analysis of costs may require variable transformation, but can be modeled using multivariate linear regression without a baseline value for costs because this value will be unknown. We will adjust for age, sex, and comorbidities that may affect cost. We recognize that we will have low statistical power to detect even moderate cost differences between the two groups.

Each nurse will provide intervention to a group of patients, so we will also measure the effect of individual nurses on outcome by multilevel analysis to determine if there is a significant variation across nurses that cannot be explained by random variation, also we will establish a number maximum of 10 patients per nurse to minimize this effect.

### Limitations

Control patients are allowed to visit study nurses during the study period so "contamination" of the control group is possible. This would reduce the point estimate of the intervention's effectiveness and may lead to a type II error. In other words, an effective intervention could be classified as ineffective because the observed effect size was neither statistically nor clinically significant. We will try to overcome this problem by using a large sample size (n = 320) based on the assumption that there will be a 15% contamination rate.

Clinical inertia is an important factor associated with poor BP control [7]. As relevant to our study, physicians of the intervention patients may be more prone to change medications if they know that their patients are involved in a clinical trial of BP control. The possibility of this effect will be significantly reduced by blinding physicians to patient allocation. However because patients are not blinding to treatment, physicians could easily find out patient allocation. We will retrospectively review the medication records to determine if there were differences in the number of medication changes in the two treatment arms and we will evaluate the effectiveness of the blinding by direct interview. We will control in a multivariate analysis by number of medication changes and physicians blinding (yes/no).

There will be a tendency of BP values, when selecting patient non optimally controlled, to resemble their mean. Randomized trials with a control group avoid regression toward the mean because BP values fell in both treated and placebo groups, but it fell by more in the treated group.

In most non-pharmacological randomized controlled trials where follow-up visits are part of the intervention, follow-up visits in the control group are common. Thus, an undesirable "Hawthorne effect" could mask the effects of our intervention. To avoid this problem, the effects of the intervention will be compared with a control group without protocol-stipulated follow-up visits.

The ideal outcome measures for our trial are reduction of cardiovascular events and/or total mortality. However, such a study would be very expensive because it would require a very large sample size and very long study period. The use of clinically relevant reduction of BP (a surrogate marker) allows us to perform this study with a more manageable number of patients (n = 320) in a relatively short period of time (12 months).

## Discussion

Unfortunately, patient adherence to AHT is sub-optimal, and BP levels remain above recommended guidelines for many AHT patients. Increasing the support provided to patients and investigating patients' beliefs about treatment are likely to improve adherence. Our study will investigate whether a multifactorial adherence intervention program applied in a primary care setting leads to improved control of BP. Adherence interventions could improve the effectiveness and efficacy of pharmacotherapy and could help reduce future healthcare costs associated with the long-term consequences of uncontrolled hypertension.

Recent studies of AHT [14,15] have reported that there was a significantly decreased risk of cardiovascular events in the high adherence groups (> 80%) compared with low adherence groups (< 40%).

Efforts to develop novel antihypertensive agents are expensive and likely to yield drugs whose efficacy does not differ significantly from existing blood pressure lowering agents in terms of major cardiovascular events, cardiovascular death, and total mortality [4]. By contrast, development of an effective adherence-based intervention could provide better results at a lower cost.

## Competing interests

The authors declare that they have no competing interests.

## Authors' contributions

Conception of the idea for the study: AL. Development of the protocol, organization and funding: AL, LE, FJA, SL, MCM, RG, GT, EMGarcía, RD, RO, FS, PD, JM, PL, CV, AAI and RF. Primary care clinical committee responsible for the intervention programme, treatment, adherence and monitoring: AL y AMB. Writing of the manuscript: AL, MF, AAI, CV and JLl. All the authors have read the draft critically, to make contributions, and have approved the final text.

## Pre-publication history

The pre-publication history for this paper can be accessed here:

http://www.biomedcentral.com/1471-2261/10/44/prepub
